# Analgesic efficacy and safety of erector spinae versus serratus anterior plane block in thoracic surgery: a systematic review and meta-analysis of randomized controlled trials

**DOI:** 10.1186/s44158-023-00138-y

**Published:** 2024-01-12

**Authors:** Qurat Ul Ain Muhammad, Muhammad Ahmad Sohail, Noor Mahal Azam, Hafiza Hifza Bashir, Hira Islam, Rana Ijaz, Sakina Aquil, Tehreem Mansoor, Bishal Dhakal, Tehniat Fatima, Javeria Noor, Alina Sami Khan, Arham Iqbal, Mahima Khatri, Satesh Kumar

**Affiliations:** 1 Rawalpindi Medical University, Chamanzar Colony, Tipu Road, Rawalpindi, 46000 Pakistan; 2 Shifa College of Medicine, Islamabad, Pakistan; 3 Liaquat National Hospital and Medical College, Karachi, Pakistan; 4 Karachi Medical and Dental College, Karachi, Pakistan; 5 Services Institute of Medical Sciences, Lahore, Pakistan; 6 Dow University of Health Sciences, Karachi, Pakistan; 7 Nepalese Army Institute of Medical Sciences, Kathmandu, Nepal; 8 King Edward Medical University, Lahore, Pakistan; 9 Shaheed Mohtarma Benazir Bhutto Medical College, Karachi, Pakistan

**Keywords:** Regional anesthesia, Nerve Block (D009407), Post-operative Pain (D010149), Thoracotomy (D013908), Thoracic Surgery, Video-Assisted (D020775)

## Abstract

**Background:**

Erector spinae plane block (ESPB) and serratus anterior plane block (SAPB) are regional anesthesia techniques that have shown favorable results in pain management following thoracic surgeries; however, their relative superiority is unclear. This review (PROSPERO: CRD42023443018) aims to compare the analgesic efficacy of ESPB and SAPB in patients undergoing thoracic surgeries through the pooled analysis of co-primary outcomes: postoperative oral-morphine-equivalent (mg) consumption in 24 h and pain scores (static) at 24 h.

**Methods:**

A literature search was conducted across PubMed, Cochrane Library, and Google Scholar to identify randomized controlled trials (RCTs) from inception to May 2023, comparing ESPB and SAPB in thoracic surgeries. Statistical pooling was done using Review Manager 5.4.1. Bias assessment employed the Cochrane Collaboration Risk-of-Bias 2.0 tool. The strength of evidence was assessed using the guidelines from the GRADE working group.

**Results:**

Nine RCTs (485 patients) were included in the study. Postoperative pain scores (static) at 24 h (mean difference (MD) =  − 0.31 [− 0.57, 0.05], *p* = 0.02) and postoperative oral-morphine-equivalent (mg) consumption in 24 h (MD =  − 19.73 [− 25.65, − 13.80], *p* < 0.00001) were significantly lower in the ESBP group. However, the MDs did not exceed the set threshold for clinical importance. No significant differences were observed in the opioid-related adverse effects and block-related complications.

**Conclusion:**

Our statistically significant results imply that ESPB has superior analgesic efficacy compared to SAPB; however, this difference is clinically unimportant. The safety profile of the two blocks is comparable; hence, current evidence cannot define the relative superiority of one block over the other. Our findings warrant further research with standardized methodologies and a longer duration of analgesic efficacy assessment to yield robust evidence for better clinical applications.

**Supplementary Information:**

The online version contains supplementary material available at 10.1186/s44158-023-00138-y.

## Introduction

Thoracic surgical procedures involving thoracotomy and video-assisted thoracoscopic surgery (VATS) are associated with significant postoperative pain of varying intensity and duration [1, 2]. Despite advancements in medical practice, adequate postoperative pain management remains elusive [3]. Post-thoracotomy pain originating from pleural irritation and damage to the intercostal nerves and muscles is one of the most severe postsurgical pains [4]. It is associated with significant morbidity due to decreased mobility, impaired respiratory function, hemodynamic instability, and chest infections [5, 6]. Adequate management of acute pain is crucial to prevent these complications and to avert persistent postsurgical pain (PPSP), which develops in about 25 to 60% of patients [3]. Postoperative opioids have traditionally been used to alleviate pain; however, their limitations have necessitated exploring alternative approaches such as thoracic epidural analgesia (TEA), paravertebral block (PVB), interfascial plane blocks, and intercostal nerve blocks [7].

Serratus anterior plane block (SAPB) and erector spinae plane block (ESPB), two interfascial plane blocks, described by Blanco et al. in 2013 and Forero et al. in 2016, have demonstrated pain relief in thoracic surgeries. These blocks gained popularity due to their efficacy and favorable adverse effect profile [8]. SAPB acts on the lateral branches of the intercostal nerves, blocking pain reception in the chest wall [9], while ESPB involves the deposition of local anesthetic (LA) in the fascial plane between erector spinae muscle and tips of the transverse processes of the vertebrae [10–12]. To prevent excessive opioid consumption [13, 14] and reduce complications stemming from severe postoperative pain, it is imperative to provide optimal regional anesthesia in accordance with the enhanced recovery after surgery (ERAS) protocol [15].

This systematic review and meta-analysis aim to address the existing knowledge gap regarding the comparative effectiveness of the two blocks by synthesizing robust and reliable evidence. Notably, to our knowledge, this specific topic has not yet been the subject of a meta-analysis, and existing randomized controlled trials (RCTs) have reported conflicting evidence [12, 15], making our research particularly valuable in providing a comprehensive synthesis of existing evidence on these analgesic approaches. The primary objective is to identify which technique, ESPB or SAPB, provides superior analgesic efficacy in terms of pain severity at rest and opioid consumption during the postoperative period, particularly within the first 24 h.

## Methods

Preferred Reporting Items for Systematic Reviews and Meta-Analyses statement (PRISMA) guidelines were followed for this systematic review and meta-analysis [16]. The protocol for this meta-analysis was registered in the International Prospective Register for Systematic Reviews (PROSPERO) with ID CRD42023443018. The study protocol was modified to exclude observational studies and studies comparing the efficacy of the two blocks in patients undergoing breast surgery.

### Eligibility criteria

We included RCTs that met the following criteria: (1) assessed the use of ESPB as intervention and SAPB as a comparator, (2) involved patients undergoing thoracic surgery (thoracotomy or VATS), (3) reported postoperative pain scores at 24 h or postoperative opioid consumption within 24 h as outcomes, and (4) provide full-text access, either in English or any other language. Additionally, articles that did not provide the data necessary for calculating a mean difference (MD) and a 95% confidence interval (CI) were excluded.

### Search strategy

The authors conducted a systematic literature review on PubMed, Google Scholar, and Cochrane Library from inception through July 2023, using a preformulated search term to retrieve all pertinent publications. The search string was generated by combining keywords related to the following terms: SAPB, ESPB, thoracic surgery, thoracotomy, and VATS. Online supplementary appendix A provides the detailed search technique. Additionally, the bibliography of potentially eligible articles was examined for relevant studies.

EndNote X7 was used to store references and remove any duplicate studies. Initially, two impartial reviewers [S.K. and Q.M.] skimmed the titles and abstracts, and a third reviewer [H.H.B.] was brought in in case of disparities. Finally, the entire text was read thoroughly to determine eligibility.

### Data extraction

The data extraction team [NMA and MAS] created an extraction form on Google Sheets. The retrieved data included the name of the first author, year of publication, total number of participants, mean age, gender, and BMI of participants; type of surgery; details of the block procedure and analgesic regimen used; primary outcomes (rest pain scores at 24 h and postoperative opioid consumption in 24 h); and secondary outcomes (rest pain scores at 2 and 12 h, pain scores on movement at 2, 12, and 24 h, time to receive the first dose of postoperative opioids, successful-block in the first attempt, adverse effects including nausea vomiting, itching and hypotension). For continuous outcome data, we extracted the mean (standard deviation). Standardized statistical conversions were made if the data was reported as median (IQR). Dichotomous data was extracted in events/total format. Graphical data was extracted using the Plot digitizer online application. Extracted data was verified by a third reviewer [HI].

### Quality assessment and risk of bias

To evaluate the methodological quality of the RCTs, we employed the Cochrane Collaboration Risk-of-Bias 2.0 tool [17], which comprises five domains. The tool assesses bias arising from randomization, deviation from planned intervention, missing data, inappropriate outcome measurement methods, and selective reporting. Two independent reviewers [Q.M. and R.I.] meticulously assessed each trial’s methodology and assigned a risk of bias rating as low, unclear, or high based on predetermined criteria. Any discrepancies were resolved through reevaluation by a third reviewer [S.A.].

For each statistically pooled outcome, we assessed the overall strength of evidence using the guidelines created by the Grades of Recommendation, Assessment, Development, and Evaluation (GRADE) working group [18]. Following this, the evidence strength was classified as high-quality (⊕⊕⊕⊕), moderate-quality (⊕⊕⊕⊕ ⊖), low-quality (⊕⊕ ⊖ ⊖), or very low-quality (⊕ ⊖  ⊖ ⊖) evidence.

### Primary and secondary outcomes

The co-primary outcomes included postoperative pain scores (static) at 24 h and postoperative oral morphine (mg) equivalent consumption at 24 h. The secondary outcomes we evaluated were postoperative pain scores (static) at 2 and 12 h, pain scores (dynamic) at 2, 12, and 24 h, time to receive the first dose of postoperative opioids, successful block in the first attempt, opioid-related adverse effects, and block-related complications.

### Measurement of outcomes

Postoperative pain scores were recorded at rest (static) and on movement (dynamic) at 2, 12, and 24 h. The pain score data was converted into an equivalent score on a 0–10 cm visual analog scale (VAS: 0: no pain; 10: worst experienced pain). The doses of different postoperative opioids consumed within 24 h were converted into equivalent doses of oral morphine in milligrams using a standardized converter [19]. All the time-to-event data was converted to hours.

### Interpretation

We interpreted our co-primary outcomes in terms of minimal clinically important difference (MCID). The outcome “postoperative pain scores (static) at 24 h” was deemed clinically important if the mean difference (MD) of the pooled VAS score exceeded 1.1 cm [20]. Similarly, the MCID for “24-h postoperative oral morphine (mg) equivalent consumption” was taken as 30 mg of oral morphine [20].

### Statistical analysis

Review Manager 5.4.1 was used for meta-analysis. The generic-inverse variance method with a random-effects model was used to calculate the MD with the corresponding 95% confidence interval (CI) for continuous variables. For dichotomous variables, the Mantel–Haenszel method with a random-effects model was used to calculate the risk ratio (RR). *p* < 0.05 was defined as the threshold for statistical significance.

The results of the pooled studies were demonstrated in forest plots, and funnel plots were created to evaluate publication bias. To confirm our findings, Egger’s regression test was applied. Higgin’s *I*^2^ test was used to assess the degree of inconsistency among the included studies. The degree of heterogeneity was defined as follows: low heterogeneity (*I*^2^ < 25%), moderate (*I*^2^ = 25–75%), and high (*I*^2^ > 75%). Moderate and high heterogeneity necessitated the exploration of the causes of heterogeneity [21].

### Methods to explore causes of heterogeneity

Sensitivity analysis, subgroup analysis, and meta-regression were performed to explore reasons for significant heterogeneity. Sensitivity analysis was performed by the e sequential exclusion of the studies on the basis of (1) the use of perineural adjuncts like epinephrine, dexamethasone, or lignocaine in the LA mixture; (2) the use of a continuous infusion of LA instead of a single-shot block; (3) the use of any LA other than the most commonly used LA, i.e., bupivacaine; and (4) administration of block before general anesthesia. To perform subgroup analysis, the studies were divided into two subgroups according to the type of surgery performed: thoracotomy and VATS. A univariate meta-regression was performed using STATA 17.0 to identify study-level variables that might have been a possible source of substantial heterogeneity across the results of the two primary outcomes. The dose of the LA and the mode of postoperative analgesia (unimodal = purely opioid-based vs. multimodal = use of analgesic adjuvants such as paracetamol, and non-steroidal anti-inflammatory drugs) were identified as potential sources of high heterogeneity. Previous reviews comparing nerve blocks have also attributed high heterogeneity to the above co-variates [20, 22]. A meta-regression coefficient(β) was considered statistically significant at *p* ≤ 0.05.

## Results

### Study selection

**Fig. 1:**
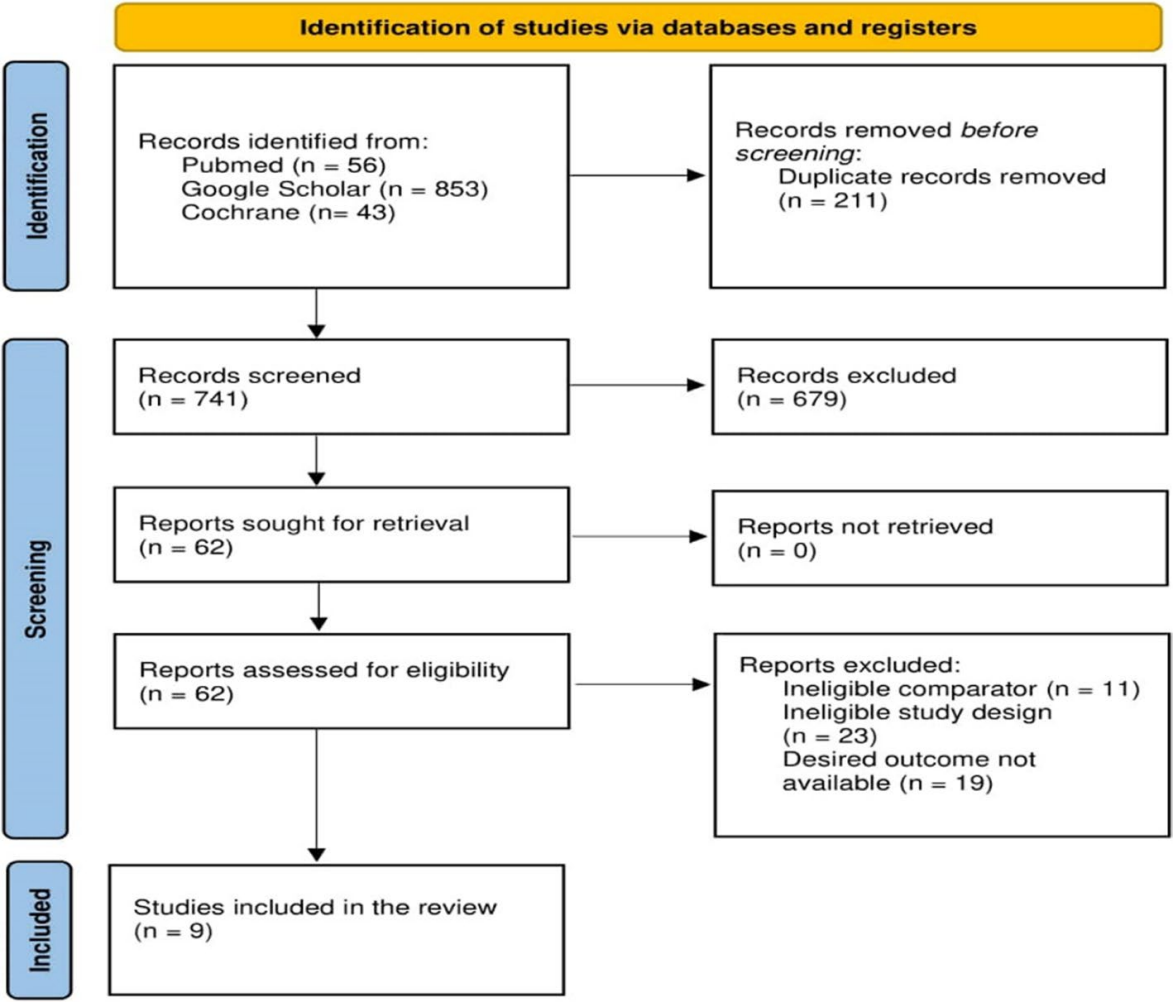
PRISMA Flowchart. *PRISMA* Preferred Reporting Items for Systematic Reviews and Meta-Analyses

The search strategy yielded 588 results. After removing duplicates, a total of 377 citations were subject to title and abstract screening. Of these, 315 were excluded, leaving 62 studies for full-text examination. Finally, 9 RCTs [7, 12, 15, 23–28] were included in the meta-analysis (Fig. 1).

### Risk of bias assessment

**Fig. 2:**
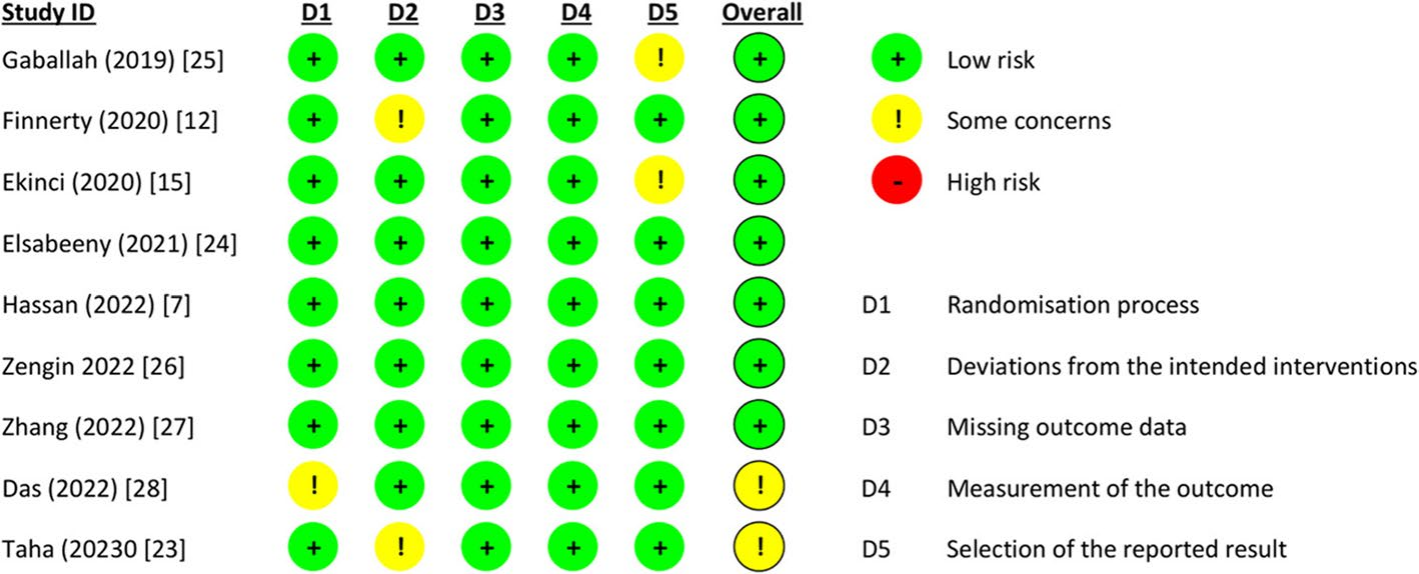
Risk of bias assessment for the included trials using Cochrane risk of bias tool 2.0

Eight studies [7, 12, 15, 23–26] adequately described the random sequence generation methods and reported using allocation concealment to reduce bias. Three studies [7, 12, 26] clearly stated that the participants were blinded. Three studies [7, 24, 26] explicitly reported that the outcome assessors were also blinded. All the studies were low risk for reporting bias (Fig. 2).

### Study characteristics

The included studies were conducted in various countries, such as Turkey, Egypt, Ireland, India, Italy, Belgium, and the USA, from 2019 to 2022. The number of participants in the studies ranged from 34 to 60. Three studies included patients undergoing thoracotomy [7, 24, 28], while six studies included patients undergoing VATS [12, 15, 23, 25, 26] (online supplementary appendix B). The details of block characteristics and analgesic regimes have been summarized in Table 1. Regarding the LA used, five studies [7, 15, 23, 25, 26] used 20 mL of 0.25–0.5% bupivacaine, while three studies used 30 mL of 0.25% bupivacaine [24, 28] and levobupivacaine [12]. Only one study used 20 mL of 0.4% ropivacaine [26].

**Table 1:** Details of block procedure and analgesic regimens in the included RCTs

**Author/Year**	**Preincisional analgesia and anti-emetics**	**Block timing**	**ESPB local anesthetic bolus**	**SAPB local anesthetic bolus**	**Localization**	**Supplemental postoperative analgesia**
Gaballah (2019) [25]	Lidocaine 1% (5 ml/port site)	Preoperative (after GA)	20 mL of 0.25% bupivacaine	20 ml of 0.25% bupivacaine superficial to SAM	USG	If VAS ≥ 4, IV ketorolac 30 mg, IV pethidine 0.5 mg/kg prn
Finnerty (2020) [12]	Paracetamol 1 g, IV and dexketoprofen 50 mg, ondansetron 0.1 mg /kg, Dex 0.1 mg/kg	Preoperative (after GA)	30 ml of 0.25% Levobupivacaine in	levobupivacaine 0.25% in 30 ml volume deep to the SAM	USG	Oxycodone 2 mg IV until VAS ≤ 2, paracetamol 1 g IV 6 hourly, ibuprofen 400 mg orally eight hourly, ondansetron 4 mg PO/IV q8h
Ekinci (2020) [15]	-	Preoperative (before GA)	20 mL volume of 0.25% bupivacaine	20-mL volume of 0.25% bupivacaine deep to SAM	USG	400 mg ibuprofen and a 100 mg tramadol IV immediately after the procedure, ibuprofen 400 mg IV q8h, PCA 2 ml of 10-mg/mL fentanyl, lockout time of 20 min, meperidine (0.5 mg/kg) IV if VAS ≥ 4
Elsabeeny (2021) [24]	1 g paracetamol IV infusion	Preoperative, single shot (after GA) followed by continuous infusion for 24 h postoperatively	30 mL of 0.25% bupivacaine followed by continuous infusion of 0.125% bupivacaine at a rate of 8–10 mL/h	30 mL of 0.25% bupivacaine deep to SAM, followed by continuous infusion of 0.125% bupivacaine at the rate of 8–10 mL/h	USG	1 g paracetamol IV infusion q8h, if VAS < 4, IV 30 mg ketorolac given; if VAS ≥ 4, IV morphine with a maximum dose of 0.1 mg/kg
Hassan (2022) [7]	-	Preoperative (before GA)	20 ml of 0.5% bupivacaine	20 ml of 0.5% bupivacaine superficial to SAM	USG	PCIA: 1 mg morphine of 1 mg/ml solution lockout interval of 10 min
Zengin 2022 [26]	IV 50 mg dexketoprofen, 100 mg tramadol	Preoperative (after GA)	20 mL of 0.25% bupivacaine	10 mL of 0.25% bupivacaine deep and superficial to SAM	USG	PCIA: 1 mg morphine with a lockout interval of 15 min. IV 1 g paracetamol q8h, 50 mg dexketoprofen twice daily
Zhang (2022) [27]	IV sufentanil 0.5 μg/kg	Preoperative (after GA)	20 mL of 0.4% ropivacaine	20 mL of 0.4% ropivacaine deep to SAM	USG	PCIA: 150 μg sufentanil with a loading dose of 2 mL, background dose of 2 mL, and locking duration of 15 min. IV 40 mg parecoxib sodium until NRS ≤ 3
Das (2022) [28]	-	Preoperative (before GA)	30 mL of LA mixture (bupivacaine 0.5% and 2% lignocaine with adrenaline 1:100,000)	30 mL of LA mixture (bupivacaine 0.5% and 2% lignocaine with adrenaline 1:100,000) superficial to SAM	USG	PCIA: 0.5 μg/kg fentanyl
Taha (20,230 [23]	-	Preoperative (after GA)	20 mL of 0.25% bupivacaine	20 mL of 0.25% bupivacaine superficial to SAM	USG	IV 1 g paracetamol q8h for the first 24 h, if VAS ≥ 3, IV pethidine 25 mg with maximum dose 150 mg/day

*GA* general anesthesia, *ESPB* erector spinae plane block, *SAPB* serratus anterior plane block, *LA* local anesthetic, *USG* ultrasound-guided, *IV* intravenous, *q* every, *prn* as needed, *PCIA* patient-controlled intravenous analgesia, *SAM* serratus anterior muscle, *VAS* visual analog scale, *Dex* dexamethasone.

### Outcomes

Outcomes are represented in a tabulated form in online supplementary appendix B.

### Primary outcomes

#### Postoperative pain scores (static) at 24 h

**Fig. 3:**
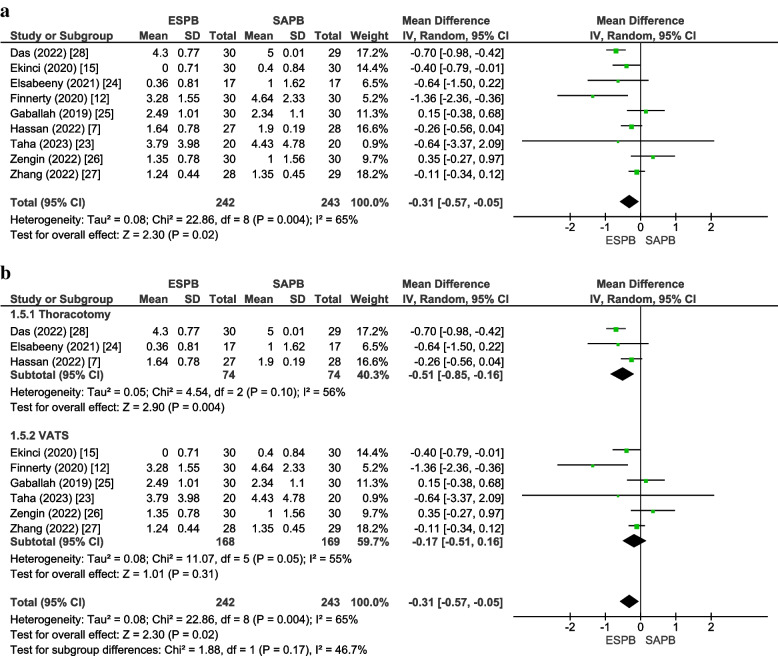
**a **Forest plot of postoperative pain scores (static) at 24 h. The MD estimates for each study are represented by squares, and the lines passing through them represent 95% CI. The diamond represents the overall pooled estimate. **b** Postoperative pain scores (static) at 24 h (forest plot of subgroup analysis). *MD* mean difference, *CI* confidence interval, *IV* inverse variance, *SD* standard deviation, *ESPB* erector spinae plane block, *SAPB* serratus anterior plane block, *VATS* video-assisted thoracoscopic surgery

Nine studies [7, 12, 15, 23–28] inclusive of 485 patients (ESPB:242, SAPB:243) reported rest pain scores at 24 h. The pooled analysis showed that patients receiving ESPB reported less pain than those given SAPB (MD =  − 0.31 [− 0.57, − 0.05], *p* = 0.02, *I*^2^ = 65%) (Fig. 3a). ESPB reduced pain score at 24 h in the subgroup thoracotomy (MD =  − 0.51 [− 0.85, − 0.16], *p* = 0.004, *I*^2^ = 56%). For the subgroup “VATS,” there was no significant difference in the pain scores between the two groups (MD =  − 0.17 [− 0.51, 0.16], *p* = 0.31, *I*^2^ = 55%) (Fig. 3b). None of the MDs reached the threshold for clinical importance. Our results were robust to sensitivity analysis; the exclusion of the studies on the basis of the use of perineural adjunct (lidocaine + epinephrine) [28] and administration of block before general anesthesia (GA) [15, 28] reduced the heterogeneity to 0%. Visually, the funnel plot appeared symmetrical, and Egger’s regression revealed a non-significant intercept (*p* = 0.77), indicating the absence of publication bias (online supplementary appendix C). Hence, the GRADE strength of evidence was high (Table 2).

**Table 2:** Evidence profile for patients receiving erector spinae vs serratus anterior plane block in thoracic surgeries

Outcomes	Limitations	Inconsistency	Indirectness	Publication bias	Mean difference or RR [95% CI]	No of participants (studies)	Strength or certainty of the evidence (GRADE)
Postoperative Pain Scores (Static) at 24 h	No serious limitation	Moderate test for inconsistency (*I*^2^ = 65); resolution on sensitivity analysis	Not detected	Not detected	− 0.31 [− 0.57, − 0.05]	485 (9)	⊕ ⊕ ⊕ ⊕ High
24-h postoperative oral morphine (mg) equivalent consumption	No serious limitation	High test for inconsistency (*I*^2^ = 90%); resolution on sensitivity analysis for subgroup “VATS”	Not detected	Not detected	− 19.75 [− 25.65, − 13.80]	425 (8)	⊕ ⊕ ⊕ ⊖ Moderate
Pain scores (static) at 2 h	No serious limitation	High test for inconsistency (1^2^ = 95%)	Not detected	Not detected	− 0.38 [− 0.94, 0.19]	425 (8)	⊕ ⊕ ⊕ ⊖ Moderate
Pain scores (static) at 12 h	No serious limitation	High test for inconsistency (1^2^ = 91%)	Not detected	Not detected	− 0.49 [− 0.94, − 0.04]	291 (5)	⊕ ⊕ ⊕ ⊖ Moderate
Pain scores (dynamic) at 2 h	No serious limitation	Moderate test for inconsistency (1^2^ = 72%)	Not detected	Not detected	− 0.53 [1.14, 0.08]	269 (5)	⊕ ⊕ ⊕ ⊖ Moderate
Pain scores (dynamic) at 12 h	No serious limitation	Moderate test for inconsistency (1^2^ = 46%)	Not detected	Not detected	− 0.72 [− 1.09, − 0.35]	345 (4)	⊕ ⊕ ⊕ ⊖ Moderate
Pain scores (dynamic) at 24 h	No serious limitation	High test for inconsistency (1^2^ = 78%)	Not detected	Not detected	− 0.64 [− 1.13, − 0.14]	439 (6)	⊕ ⊕ ⊕ ⊖ Moderate
Time to request first dose of postoperative analgesia	No serious limitation	High test for inconsistency (1^2^ = 99%)	Not detected	Not detected	2.70 [1.64, 3.77]	310 (6)	⊕ ⊕ ⊕ Moderate
Successful block on first attempt	No serious limitation	Moderate test for inconsistency (1^2^ = 41%)	Not detected	Not detected	0.92 [0.74, 1.14]	119 (2)	⊕ ⊕ ⊕ ⊖ Moderate
Postoperative nausea	No serious limitation	Low test for inconsistency (1^2^ = 0%)	Not detected	Not detected	0.94 [0.61, 1.46]	251 (5)	⊕ ⊕ ⊕ ⊕ High
Postoperative vomiting	No serious limitation	Low test for inconsistency (1^2^ = 0%)	Not detected	Significant bias detected on Egger’s regression (*p* = 0.005)	0.86 [0.56, 1.34]	251 (5)	⊕ ⊕ ⊕ ⊖ Moderate
Hypotension	No serious limitation	Moderate test for inconsistency (1^2^ = 63%)	Not detected	Not detected	1.11 [0.17, 7.04]	189 (4)	⊕ ⊕ ⊕ ⊖ Moderate

*GRADE* Grades of Recommendation, Assessment, Development and Evaluation, *VATS* Video-assisted thoracoscopic surgery, *MD* mean difference, *RR* risk ratio, *CI* confidence interval.

#### 24-h postoperative oral morphine (mg) equivalent consumption

**Fig. 4:**
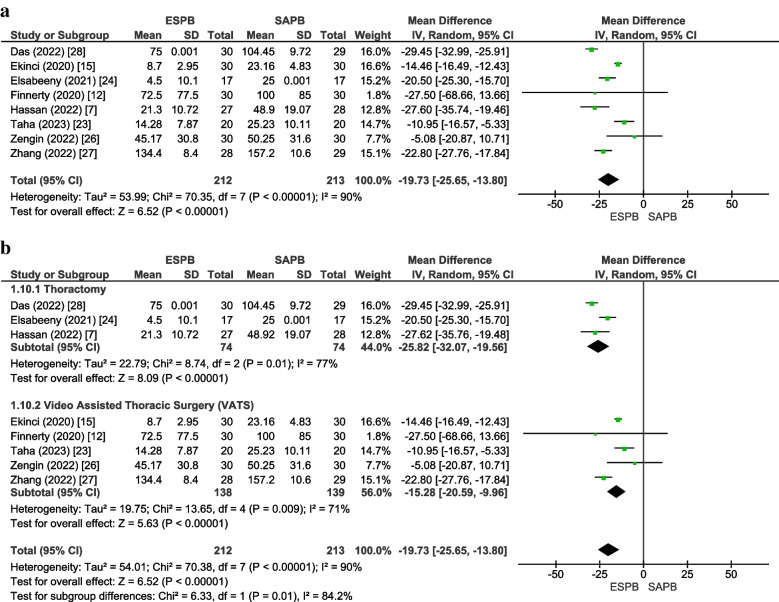
**a** Forest plot of 24-h postoperative oral morphine (mg) equivalent consumption**.** The MD estimates for each study are represented by squares and the lines passing through them represent 95% CI. The diamond represents the overall pooled estimate. **b** 24-h postoperative oral morphine (mg) equivalent consumption (forest plot for subgroup analysis). *MD* Mean difference, *CI* Confidence interval, *IV* Inverse variance, *SD* Standard deviation, *ESPB* Erector spinae plane block, *SAPB* Serratus anterior plane block, *VATS* Video-assisted thoracoscopic surgery

Eight studies [7, 12, 15, 23, 24, 26–28] inclusive of 425 patients (ESPB:212, SAPB:213) reported postoperative opioid consumption in 24 h. The pooled analysis of equivalent doses of oral morphine in milligrams revealed that the dose of morphine consumption was significantly lower in the ESPB group as compared to the SAPB group (MD =  − 19.73 [− 25.65, − 13.80], *p* < 0.00001, *I*^2^ = 90%) (Fig. 4a). The ESPB group required lower doses of postoperative opioids in the subgroup “thoracotomy” (MD =  − 25.82 [− 32.07, − 19.56], *p* < 0.00001, *I*^2^ = 77%) and “VATS” (MD =  − 15.28 [− 20.59, − 9.96], *p* < 0.00001, *I*^2^ = 71%) (Fig. 4b). Our results were statistically significant; however, the MD fell short of the threshold set for clinical importance. On sensitivity analysis, the exclusion of the study [27] based on the use of 0.4% ropivacaine reduced heterogeneity in the subgroup “VATS” to 0%. The funnel plot appeared symmetrical, and Egger’s regression intercept was insignificant (*p* = 0.68) for publication bias. The GRADE strength of the evidence was moderate (Table 2).

### Secondary outcomes

#### Pain scores (static) at 2 and 12 h

**Fig. 5:**
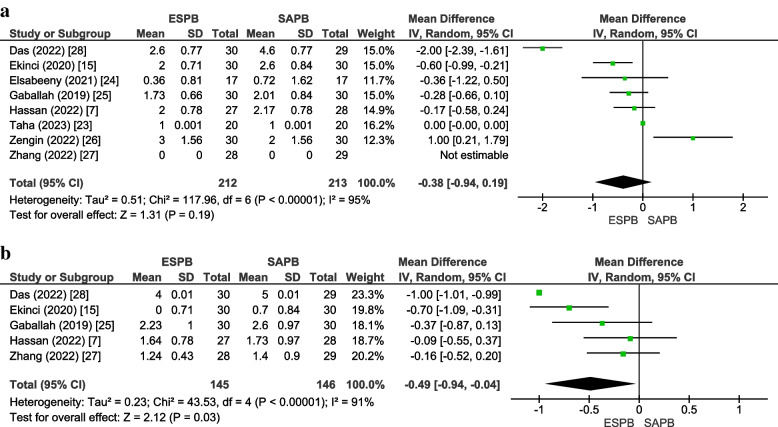
**a** Postoperative pain scores (static) at 2 h. **b** Postoperative pain scores (static) at 12 h. *MD* Mean difference, *CI* Confidence interval, *IV* Inverse variance, *SD* Standard deviation, *ESPB* Erector spinae plane block, *SAPB* Serratus anterior plane block

Eight studies [7, 15, 23–28] inclusive of 425 patients (ESPB:212, SAPB:213) reported rest pain scores at 2 h. No significant differences were observed between the two groups for this outcome (MD =  − 0.38 [− 0.94, 0.19], *p* = 0.19, *I*^2^ = 95%) (Fig. 5a). The forest plot appeared symmetrical, and Egger’s regression intercept (*p* = 0.81) was insignificant for publication bias (online supplementary appendix C).

Five studies [7, 15, 25, 26, 28] inclusive of 291 patients (ESPB:145, SAPB:146) reported rest pain scores at 12 h. The pain scores were lower in the ESPB group (MD =  − 0.49 [− 0.94, − 0.04], *p* = 0.03, *I*^2^ = 91%) (Fig. 5b). On sensitivity analysis, removing the study [28] using perineural adjuncts (lignocaine and epinephrine) reduced the heterogeneity to 43%. The funnel plot was asymmetrical, and Egger’s regression intercept (*p* = 0.02) indicated significant publication bias (online supplemental appendix C). The GRADE strength of evidence was moderate for both these outcomes (Table 2).

#### Pain scores (dynamic) at 2, 12, and 24 h

**Fig. 6:**
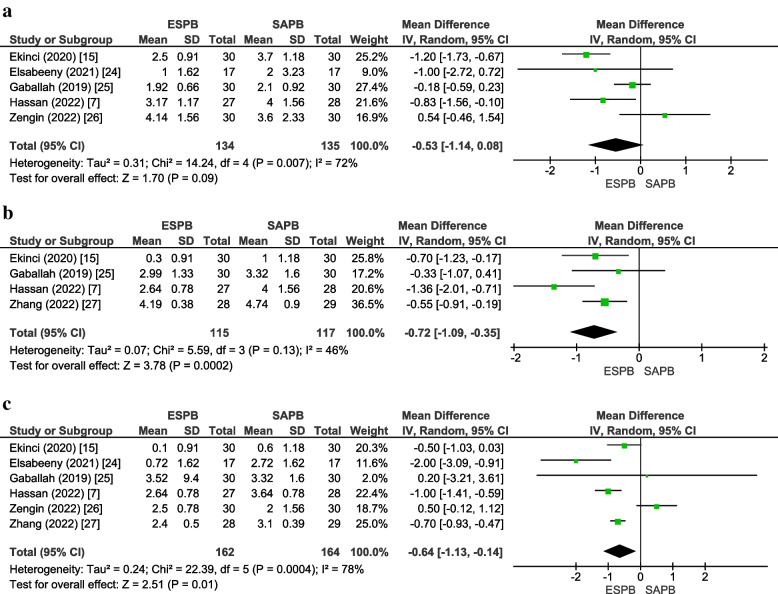
**a** Postoperative pain scores (dynamic) at 2 h. **b** Postoperative pain scores (dynamic) at 12 h. **c** Postoperative pain scores (dynamic) at 24 h. *MD* Mean difference, *CI* Confidence interval, *IV* Inverse variance, *SD* Standard deviation, *ESPB* Erector spinae plane block, *SAPB* Serratus anterior plane block

Pain scores (dynamic) for ESPB and SAPB were reported by five studies [7, 15, 24–26] at 2 h, four studies [7, 15, 25, 26] at 12 h, and six studies [7, 15, 24–26] at 24 h. There was no significant difference between the two groups at 2 h (MD =  − 0.53 [− 1.14, 0.08], *p* = 0.09, *I*^2^ = 72%) (Fig. 6a). However, pain scores were significantly lower in the ESPB group at 12 h (MD =  − 0.72 [− 1.09, − 0.35], *p* = 0.0002, *I*^2^ = 46) (Fig. 6b) and 24 h (MD =  − 0.64 [− 1.13, − 0.14], *p* = 0.01, *I*^2^ = 78%) (Fig. 6c). For all three time points, Egger’s regression intercept was insignificant for publication bias (online supplementary appendix C). The GRADE strength of evidence for all three outcomes was moderate (Table 2).

#### Time to request the first dose of postoperative analgesia (hours)

**Fig. 7:**
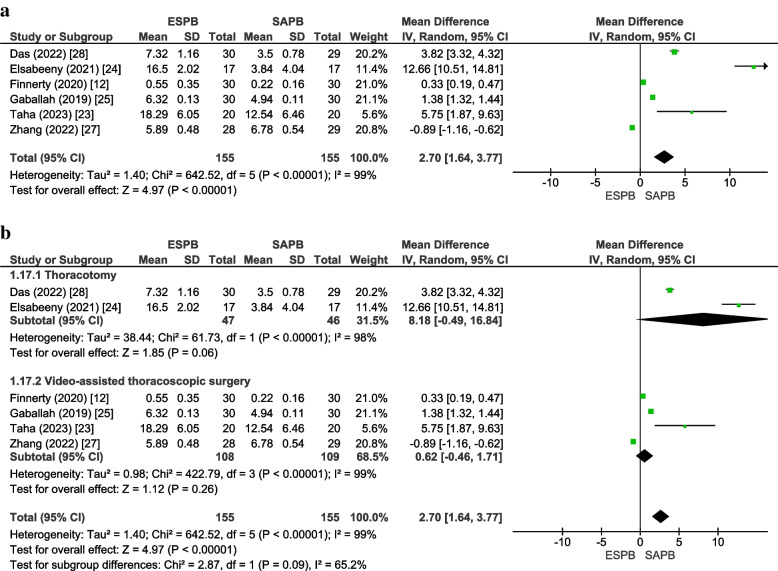
**a** Forest plot for time to request the first dose of postoperative analgesia. **b** Forest plot for time to request the first dose of postoperative analgesia (subgroup analysis). *MD* Mean difference, *CI* Confidence interval, *IV* Inverse variance, *SD* Standard deviation, *ESPB* erector spinae plane block, *SAPB* Serratus anterior plane block

Time to request the first dose of postoperative analgesia (hours) was recorded by six studies [12, 23–26, 28] with a total of 310 patients (ESPB:155, SAPB:155). The pooled analysis revealed that the time-to-first analgesic request was significantly longer in the ESBP group than in the SABP group (MD = 2.70 [1.64, 3.77], *p* < 0.00001, *I*^2^ = 99%) (Fig. 7a). Substantial heterogeneity was observed across the included studies, which was neither resolved on subgroup analysis nor sensitivity analysis. On subgroup analysis, there was no significant difference in the time to request the first dose of postoperative analgesia (Fig. 7b). Asymmetry was detected on visual inspection of the funnel plot; however, Egger’s regression intercept (*p* = 0.50) was insignificant for publication bias (online supplementary appendix C). The GRADE strength of evidence was moderate (Table 2).

#### Successful block on the first attempt

**Fig. 8:**
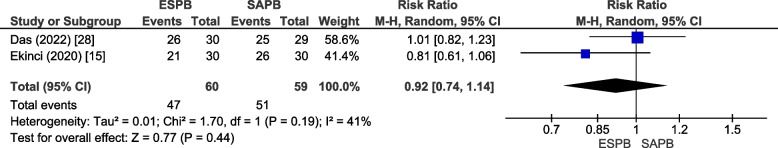
Forest plot for the successful block in the first attempt. *RR* Relative risk, *CI* Confidence interval, M–H Mantel Haenszel, *SD* Standard deviation, *ESPB* Erector spinae plane block, *SAPB* Serratus anterior plane block

Two studies [15, 28] with a total of 119 patients (ESPB:60, SAPB:59) reported the percentage of successful block administration in the first attempt (ESPB = 78.3%, SAPB = 86.4%). The pooled analysis depicted that there are no differences in the one-time puncture success rate between the ESPB group and the SAPB group (RR = 0.92 [0.74, 1.14], *p* = 0.44, *I*^2^ = 41%) (Fig. 8).

#### Opioid-related adverse effects

##### Postoperative nausea and vomiting (PONV)

**Fig. 9:**
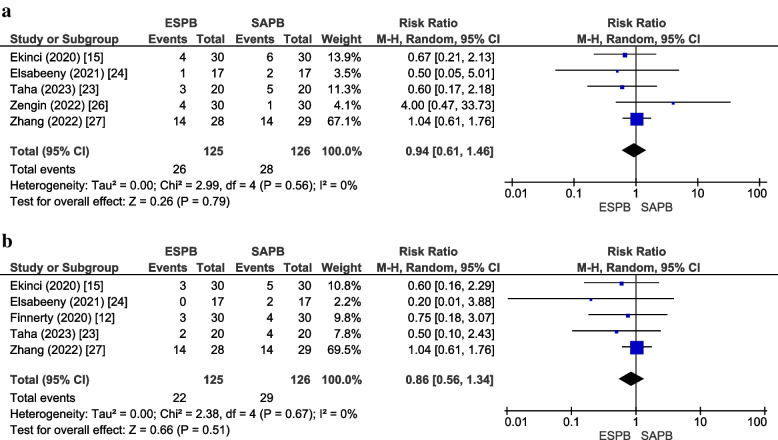
**a** Forest plot for postoperative nausea (adverse effects of the blocks). **b** Forest plot for postoperative vomiting (adverse effects of the blocks). *RR* Relative risk, *CI* Confidence interval, M–H Mantel Haenszel, *SD* Standard deviation, *ESPB* Erector spinae plane block, *SAPB* Serratus anterior plane block

Five studies [15, 23, 24, 26, 27], totaling 251 patients (ESPB:125, SAPB:126), assessed the adverse effect: nausea. 20.8% (26/125) and 22.2% (28/126) patients reported nausea in the ESPB and SAPB groups, respectively. The pooled analysis showed no significant difference. (RR = 0.94 [0.61, 1.46], *p* = 0.79, *I*^2^ = 0%) (Fig. 9a). Egger’s regression intercept was insignificant (*p* = 0.91) (online supplementary appendix C) [7, 12, 23, 24]. The GRADE strength of evidence was high (Table 2).

Five articles [12, 15, 23, 24, 27], including 251 patients (ESPB:125, SAPB:126), reported vomiting. 17.6% (22/125) and 20.3% (29/126) patients experienced vomiting in the ESPB and SAPB groups, respectively, indicating no clinically significant difference (RR = 0.86 [0.56,1.34], *p* = 0.51, *I*^2^ = 0%) (Fig. 9b). Egger’s regression intercept was significant (*p* = 0.005), indicating publication bias (online supplementary appendix C). Hence, the GRADE strength of evidence was moderate (Table 2).

#### Block-related complications

**Fig. 10:**
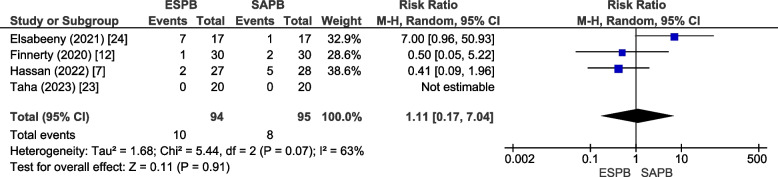
Forest plot for hypotension (adverse effects of the blocks). *RR* Relative risk, *CI* Confidence interval, *MH* Mantel Haenszel, *SD* Standard deviation, *ESPB* Erector spinae plane block, *SAPB* Serratus anterior plane block

All studies reported block-related complications. Hypotension was reported by four studies [7, 12, 23, 24], which included 189 patients. It was experienced by 10.6% (10/94) of patients in the ESPB group and 8.4% (8/95) in the SAPB group. The results were comparable among the two groups (RR = 1.11 [0.17,7.04], *p* = 0.91, *I*^2^ = 63%) (Fig. 10). Egger’s regression test (*p* = 0.79) revealed no publication bias (online supplementary appendix C). The GRADE strength of evidence was moderate (Table 2). Finnerty et al. [12] reported complications in terms of a comprehensive complication index (CCI), which was significantly lower for the ESPB group (*p* = 0.03). Zhang et al. [26] reported postoperative pneumonia and bleeding requiring transfusion with similar incidence in both groups. The remaining studies did not observe any block-related complications.

### Meta-regression

The meta-regression analysis showed that the MD of the postoperative pain scores (static) at 24 h was dependent upon the dose of LA (*β* =  − 0.06, *p* < 0.001); however, it was independent of the analgesic modality (*β* = 0.07, *p* = 0.89). The MD of the co-primary outcome, postoperative oral morphine equivalent consumption, was independent of the dose of LA (*β* =  − 0.81, *p* = 0.21) and mode of analgesia (*β* =  − 7.17, *p* = 0.41) (online supplementary appendix D).

## Discussion

### Overall findings

Our primary outcome analysis revealed statistically significant results implying that ESPB had superior analgesic efficacy. However, when these differences were interpreted in the light of MCID, none of the MDs surpassed the threshold for clinical importance. ESPB offered a modest benefit in reducing rest pain scores at 24 h by 0.31 cm (high-quality evidence) but did not reach the threshold for clinical significance, i.e., 1.1 cm [20, 29]. Similarly, ESPB reduced oral morphine consumption by 19.73 mg (moderate-quality evidence), while MCID is 30 mg of oral morphine [20]. The first-analgesic-request-time was also significantly prolonged in the ESPB group (moderate-quality evidence). Regarding the safety profile of the two blocks, there were no significant differences in the opioid-related adverse effects and block-related complications. Finnerty et al. [12] compared the block-related complications with the use of CCI [30] and reported a significantly higher score for the SAPB group. Hassan et al. [7] compared the respiratory functions among the two groups. At 24 h postoperatively, the forced vital capacity (FVC) and forced expiratory volume in the first second (FEV1) were significantly higher in the ESPB group (*p* < 0.001). These observations, despite being interesting, were recorded by only one study; hence, they merit further research and discussion. Altogether, our findings cannot define the relative superiority of one block over the other in the light of comparable efficacy and adverse effect profiles.

### Implications for research

This is the first review conducted to investigate the comparative effectiveness of the two blocks. Eight RCTs included in the review reported better analgesic efficacy of ESPB; however, Zengin et al. [26] concluded that combined deep and superficial SAPB (cSAPB) had similar efficacy to ESPB. The authors pointed out that a multi-site injection can increase the LA diffusion area and compensate for block failure in one area [26]. However, based on a pooled data analysis from existing RCTs, our review concludes that the difference between the two blocks, although statistically significant, is clinically unimportant. Future trials should assess analgesic efficacy over longer postoperative duration, i.e., 48 to 72 h. Additionally, among the included RCTs, only one study used a continuous block [24]. Future studies comparing the efficacy of single-shot versus continuous ESPB and SAPB can also provide valuable evidence as safer alternatives to TPVB and TEA.

### Anatomical perspective

ESPB and SAPB are both interfascial plane blocks of the chest wall, but our theoretical results, which imply that ESPB provides better postoperative analgesia in patients undergoing thoracic surgery, can be explained by the observation that ESPB blocks both dorsal and ventral rami of the thoracic spinal nerves in addition to some sympathetic blockade [12], while SAPB only targets the lateral cutaneous branches of the intercostal nerve. Moreover, due to its superficial nature, SAPB fails to effectively manage visceral pleural pain, particularly in pleural decortication procedures [6, 7]. Erector spinae (ES) fascia runs from the nuchal fascia to the sacrum; hence, ESPB offers a multilevel dermatomal block that can manage pain from the anterior, lateral, and posterior chest walls [9].

### Strengths and limitations

To the best of our knowledge, a comparison of ESPB and SAPB for postoperative analgesia following thoracic surgery has not yet been the subject of a meta-analysis. Our detailed search strategy identified both English and non-English studies to be included in the review. This allowed us to include nine RCTs with a total of 485 participants from various countries and ethnic groups. Thus, it has a better chance of extrapolating to the entire population. The interpretation of our results in accordance with MCID prevents the overestimation of the statistically significant differences. We used suitable methods to resolve heterogeneity. Moderate heterogeneity (*I*^2^ = 65%) was observed in the primary outcome “postoperative pain scores” (static) at 24 h. Sensitivity analysis resolved heterogeneity in our results, which could be attributed to (1) the use of perineural adjuncts in the LA mixture [28] and the administration of block before GA [15, 28]. Meta-regression revealed that the results were also dependent upon the dose of LA. High heterogeneity was observed in the co-primary outcome, which was a 24-h postoperative oral morphine (mg) equivalent consumption. The high inconsistency in results was robust to sensitivity analysis. On the exclusion of the study, Zhang et al. [26], based on the use of a different LA, i.e., ropivacaine, the heterogeneity reduced to 0% in the subgroup VATS. Conversely, there are certain limitations to this study:

- Despite sensitivity analysis, subgroup analysis, and meta-regression some residual heterogeneity remained unexplained. The diversity of the surgical procedures and anesthetic techniques could be a potential source of heterogeneity.
- Secondly, most of the studies included in this systematic review and meta-analysis had relatively small sample sizes, potentially limiting the external validity of the results. The small sample sizes also prevented us from estimating some of the rare but important block-related complications.

Despite the aforementioned shortcomings, our study remains the most current and thorough meta-analysis.

## Conclusions

Our review of nine RCTs revealed that ESPB significantly reduced rest pain scores at 24 h and decreased postoperative opioid consumption compared to SAPB in patients undergoing thoracic surgeries; however, this difference remained clinically unimportant. The safety profile of the two blocks was comparable; hence, current evidence cannot define the relative superiority of one block over the other. Our findings warrant further research with standardized methodologies and a longer duration of analgesic efficacy assessment to yield robust evidence for better clinical applications.

### Supplementary Information

**Additional file 1.**  Search strategy table. **Table S1.** Demographic characteristics of included participants. **Table S2.** Conversion of opioid consumption doses in 24 h to oral morphine (mg) equivalent doses. **Table S3.** Coprimary outcomes of the included studies. **Table S4.** Secondary outcomes of the included studies. **Fig. S1.** Funnel plots of coprimary and secondary outcomes. **Table S1.** Egger’s regression. **Table S1.** Meta-regression of coprimary outcomes

## Data Availability

The datasets supporting the conclusions of this article are included within the article and its additional files.

## Abbreviations

ESPB: Erector spinae plane block
SAPB: Serratus anterior plane block
VATS: Video-assisted thoracoscopic surgery
PPSP: Persistent postsurgical pain
TEA: Thoracic epidural analgesia
TPVB: Thoracic paravertebral block
ERAS: Enhanced recovery after surgery
RCT: Randomized controlled trial
LA: Local anesthetic
VAS: Visual analog scale
MCID: Minimal clinically important difference
MD: Mean difference
RR: Risk ratio
CI: Confidence interval
GRADE: Grades of Recommendation, Assessment, Development and Evaluation
FVC: Forced vital capacity
FEV1: Forced expiratory volume in the first second
CCI: Comprehensive complication index
