# Supplementary material for: Analgesic efficacy and safety of erector spinae versus serratus anterior plane block in thoracic surgery: a systematic review and meta-analysis of randomized controlled trials
Source: J Anesth Analg Crit Care. 2024 Jan 12;4:3. doi: 10.1186/s44158-023-00138-y (PMC10785351; doi:10.1186/s44158-023-00138-y)
Supplement: Supplementary file 1 — Additional file 1. Search strategy table. Table S1. Demographic characteristics of included participants. Table S2. Conversion of opioid consumption doses in 24 h to oral morphine (mg) equivalent doses. Table S3. Coprimary outcomes of the included studies. Table S4. Secondary outcomes of the included studies. Fig. S1. Funnel plots of coprimary and secondary outcomes. Table S1. Egger’s regression. Table S1. Meta-regression of coprimary outcomes [file 44158_2023_138_MOESM1_ESM.zip › Online Supplementary Appendix A.docx]

**ONLINE SUPPLEMENTARY APPENDIX A**

| **Database** | **Search Strategy** | **Results** |
| --- | --- | --- |
| PubMed | ((((erector spinae plane block) OR (ESPB)) OR (ESP)) AND ((((((serratus anterior plane block) OR (serratus plane block)) OR (serratus anterior block)) OR (SAPB)) OR (superficial serratus anterior plane block)) OR (deep serratus anterior plane block))) AND ((((Thoracic Surgery) OR (Thoracotomy)) OR (Thoracoscopy)) OR (Thoracic Surgery, Video-Assisted)) | 56 |
| Google Scholar | (Erector spinae plane block) AND (serratus anterior plane block) AND (Thoracotomy OR Thoracoscopy, OR Thoracic OR Thoracic Surgery Video-assisted) | 853 |
| Cochrane Library | #1 erector spinae plane block  #2 serratus anterior plane block  #3 thoracic surgery  #4 thoracotomy  #5 video-assisted thoracic surgery  #6 #3 OR #4 OR #5  #7 #1 AND #2 AND #6 | 43 |

**Search Strategy Table**

MeSH: Medical education subject heading, ESBP: Erector spinae plane block, SAPB: Serratus anterior plane block
