# Supplementary material for: Analgesic efficacy and safety of erector spinae versus serratus anterior plane block in thoracic surgery: a systematic review and meta-analysis of randomized controlled trials
Source: J Anesth Analg Crit Care. 2024 Jan 12;4:3. doi: 10.1186/s44158-023-00138-y (PMC10785351; doi:10.1186/s44158-023-00138-y)
Supplement: Supplementary file 1 — Additional file 1. Search strategy table. Table S1. Demographic characteristics of included participants. Table S2. Conversion of opioid consumption doses in 24 h to oral morphine (mg) equivalent doses. Table S3. Coprimary outcomes of the included studies. Table S4. Secondary outcomes of the included studies. Fig. S1. Funnel plots of coprimary and secondary outcomes. Table S1. Egger’s regression. Table S1. Meta-regression of coprimary outcomes [file 44158_2023_138_MOESM1_ESM.zip › Online Supplementary Appendix B.docx]

**ONLINE SUPPLEMENTARY APPENDIX B**

| **Study Name**  **and Year** | **Study**  **Type** | **Total**  **(ESPB**  **/SAPB)** | **Age ± Years** | | **Gender**  **(M/F)** | | **Body Mass Index**  **(kg/m^2^)** | | **Type**  **of Surgery** |
| --- | --- | --- | --- | --- | --- | --- | --- | --- | --- |
|  |  |  | **ESPB** | **SAPB** | **ESPB** | **SAPB** | **ESPB** | **SAPB** |  |
| Gaballah (2019) [25] | RCT | 60  (30/30) | 41.2±8.5 | 41.53±8.9 | 18/12 | 22/8 | 28.1±1.6 | 28.3±1.6 | VATS |
| Finnerty (2020) [12] | RCT | 60  (30/30) | 58.8±13 | 53.1±20 | 19/11 | 18/12 | 28.6±9.5 | 25.9±7.4 | VATS |
| Ekinci (2020) [15] | RCT | 60  (30/30) | 45.6±9.7 | 45.9± 10.3 | 14/16 | 12/18 | 26.1 | 26.7 | VATS |
| Elsabeeny (2021) [24] | RCT | 34  (17/17) | 41.9±18.7 | 42.7±17.4 | 12/5 | 10/7 | 29.25±1.8 | 28.9±2.7 | Thoracotomy |
| Taha (2023) [23] | RCT | 40  (20/20) | 45.15±9.4 | 49.07±6.3 | 11/9 | 13/7 | - | - | VATS |
| Das (2022) [28] | RCT | 59  (30/29) | 40.8 ±15.2 | 53.4±10.7 | 21/9 | 21/8 | - | - | Thoracotomy |
| Hassan (2022) [7] | RCT | 55  (27/28) | 50.3±11.8 | 48.3±9.9 | 20/7 | 17/11 | - | - | Thoracotomy |
| Zhang (2022) [27] | RCT | 50  (28/29) | 55.6±12.9 | 61.7±6.8 | 11/17 | 7/22 | 23.8±4.0 | 23.4±3.5 | VATS |
| Zengin (2022) [26] | RCT | 60  (30/30) | - | - | 20/10 | 23/7 | 25.49±4.62 | 23.7±3.24 | VATS |

**Table: Demographic characteristics of included participants**

ESPB: Erector spinae plane block, SAPB: Serratus anterior plane block, VATS: Video-assisted thoracoscopic surgery, RCT: Randomised controlled trials, M/F: Male/Female; All values are represented in mean ± standard deviation.

| **Study** | **Opioid Used** | **Mode of Admin.** | **Opioid Consumption in 24 hours** | | **Postoperative Oral Morphine (mg) Equivalent** | |
| --- | --- | --- | --- | --- | --- | --- |
|  |  |  | **ESPB** | **SAPB** | **ESPB** | **SAPB** |
| Finnerty (2020) [12] | Oxycodone | Oral | 29±31 | 40±34 | 72.5±77.5 | 100±85 |
| Ekinci (2020) [15] | Fentanyl | IV | 34.85±11.8 | 92.65±19.3 | 8.7±2.95 | 23.16±4.83 |
| Elsabeeny (2021) [24] | Morphine | IV | 1.8±4.04 | 10±0 | 4.5±10.1 | 25±0 |
| Taha (2023) [23] | Pethidine | IV | 35.71±19.67 | 63.08±25.29 | 14.28±7.87 | 25.23±10.11 |
| Das  (2022) [28] | Fentanyl | IV | 300±0.001 | 417.8±38.9 | 75±0 | 104.45±9.72 |
| Hassan (2022) [7] | Morphine | IV | 8.52±4.29 | 19.57±7.63 | 21.3±10.72 | 48.92±19.07 |
| Zhang (2022) [27] | Sufentanil | IV | 67.2±4.2 | 78.6±5.3 | 134.4±8.4 | 157.2±10.6 |
| Zengin (2022) [26] | Morphine | IV | 18.07±12.32 | 20.1±12.64 | 45.17±30.8 | 50.25±31.6 |
|  | | | | | | |

**Table: Conversion of opioid consumption doses in 24 hours to oral morphine (mg) equivalent doses**

ESPB: Erector spinae plane block, SAPB: Serratus anterior plane block, Admin: Administration, IV: Intravenous; All values are represented in mean ± standard deviation.

| **Study (Year)** | **Postoperative Pain (Static) at 24-hours** | | **24-hour Postoperative Oral morphine (mg) equivalent consumption** | |
| --- | --- | --- | --- | --- |
|  | **ESPB** | **SAPB** | **ESPB** | **SAPB** |
| Gaballah (2019) [25] | 2.49±1.01 | 2.34±1.1 | - | - |
| Finnerty (2020) [12] | 3.28±1.55 | 4.64±2.33 | 72.5±77.5 | 100±85 |
| Ekinci (2020) [15] | 0±0.71 | 0.4±0.84 | 8.7±2.95 | 23.16±4.83 |
| Elsabeeny (2021) [24] | 0.36±0.81 | 1±1.62 | 4.5±10.1 | 25±0.001 |
| Taha (2023) [23] | 3.79±3.98 | 4.43±4.78 | 14.28±7.87 | 25.23±10.11 |
| Das (2022) [28] | 4.3±0.77 | 5±0.01 | 75±0.001 | 104.45±9.72 |
| Hassan (2022) [7] | 1.64±0.78 | 1.9±0.19 | 21.3±10.72 | 48.9±19.07 |
| Zhang (2022) [27] | 1.24±0.44 | 1.35±0.45 | 134.4±8.4 | 157.2±10.6 |
| Zengin (2022) [26] | 1.35±0.78 | 1±1.56 | 45.17±30.8 | 50.25±31.6 |

**Table: Coprimary outcomes of the included studies**

ESPB: Erector spinae plane block, SAPB: Serratus anterior plane block; All values are represented in mean ± standard deviation.

| **Study (Year)** |  | **Gaballah**  **(2019) [25]** | **Finnerty (2020) [12]** | **Ekinci (2020) [15]** | **Elsabeeny (2021) [24]** | **Taha**  **(2023) [23]** | **Das (2022) [28]** | **Hassan (2022) [7]** | **Zhang (2022) [27]** | **Zengin (2022)**  **[26]** |
| --- | --- | --- | --- | --- | --- | --- | --- | --- | --- | --- |
| **Pain Scores (Static) at 2-hrs** | **E** | 1.73±0.66 | - | 2±0.71 | 0.36±0.81 | 1±0.001 | 2.6±0.77 | 2±0.78 | 0±0 | 3±1.56 |
|  | **S** | 2.01±0.84 | - | 2.6±0.84 | 0.72±1.62 | 1±0.001 | 4.6±0.77 | 2.2±0.78 | 0±0 | 2±1.56 |
| **Pain Scores (Static) at 12-hrs** | **E** | 2.23±1 | - | 0±0.71 | - | 0.5±0.76 | 4±0.001 | 1.6±0.78 | 1.24±0.43 | - |
|  | **S** | 2.6±0.97 | - | 0.7±0.84 | - | 0.4±0.76 | 5±0.001 | 1.7±0.97 | 1.4±0.9 | - |
| **Pain Scores (Dynamic) at 2-hrs** | **E** | 1.92±0.66 | - | 2.5±0.91 | 1±1.62 | - | - | 3.2±1.17 | 1±1.57 | 4.1±1.56 |
|  | **S** | 2.1±0.92 | - | 3.7±1.18 | 2±3.23 | - | - | 4±1.56 | 2.4±0.8 | 3.6±2.33 |
| **Postoperative Pain (Dynamic) at 12-hrs** | **E** | 2.99±1.33 | - | 0.3±0.91 | - | - | - | 2.6±0.78 | 4.19±0.38 | - |
|  | **S** | 3.32±1.6 | - | 1±1.18 | - | - | - | 4±1.56 | 4.74±0.9 | - |
| **Pain Scores (Dynamic) at 24-hrs** | **E** | 3.52±9.4 | - | 0.1±0.91 | 0.72±1.62 | - | - | 2.64±0.78 | 2.4±0.5 | 2.5±0.78 |
|  | **S** | 3.32±1.6 | - | 0.6±1.18 | 2.72±1.62 | - | - | 3.64±0.78 | 3.1±0.39 | 2±1.56 |
| **Time-to-first analgesic request** | **E** | 6.32±0.13 | 0.5±0.4 | - | 16.2±0.69 | 18.3±6.01 | 7.4±0.45 | - | 5.89±0.48 | - |
|  | **S** | 4.94±0.11 | 0.2±0.2 | - | 2.8±1.39 | 12.5±6.46 | 3.5±0.8 | - | 6.78±0.54 | - |
| **Nausea *n* (%)** | **E** | - | - | 4(13.3) | 1(5.8) | 3(15) | - | - | 14(50) | 4(13.3) |
|  | **S** | - | - | 6(20) | 2(11.7) | 5(25) | - | - | 14(48.3) | 1(3.3) |
| **Vomiting *n* (%)** | **E** | - | 3(10) | 3(10) | 0(0) | 2(10) | - | - | 14(50) | - |
|  | **S** | - | 4(13.3) | 5(16.7) | 2(11.7) | 4(20) | - | - | 14(48.3) | - |
| **Hypotension *n* (%)** | **E** | - | 1(3.3) | - | 7(41.2) | 0(0) | - | 2(7.1) | - | - |
|  | **S** | - | 2(6.6) | - | 1(5.9) | 0(0) | - | 5(18.5) | - | - |
| **Successful block in**  **first attempt n (%)** | **E** | - | - | 21(70) | - | - | 26(86.6) | - | - | - |
|  | **S** | - | - | 26(86.7) | - | - | 25(86.2) | - | - | - |

**Table: Secondary outcomes of the included studies**

E: Erector spinae plane block, S: Serratus anterior plane block; Continuous outcomes are represented in mean ± standard deviation.
