# Supplementary material for: Analgesic efficacy and safety of erector spinae versus serratus anterior plane block in thoracic surgery: a systematic review and meta-analysis of randomized controlled trials
Source: J Anesth Analg Crit Care. 2024 Jan 12;4:3. doi: 10.1186/s44158-023-00138-y (PMC10785351; doi:10.1186/s44158-023-00138-y)
Supplement: Supplementary file 1 — Additional file 1. Search strategy table. Table S1. Demographic characteristics of included participants. Table S2. Conversion of opioid consumption doses in 24 h to oral morphine (mg) equivalent doses. Table S3. Coprimary outcomes of the included studies. Table S4. Secondary outcomes of the included studies. Fig. S1. Funnel plots of coprimary and secondary outcomes. Table S1. Egger’s regression. Table S1. Meta-regression of coprimary outcomes [file 44158_2023_138_MOESM1_ESM.zip › Online Supplementary Appendix C.docx]

**ONLINE SUPPLEMENTARY APPENDIX C**

| **Pain scores (static) at 2-hours**  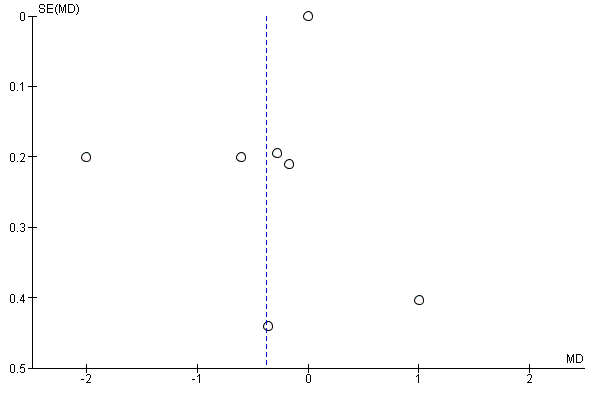 | **Pain scores (dynamic) at 2-hours**  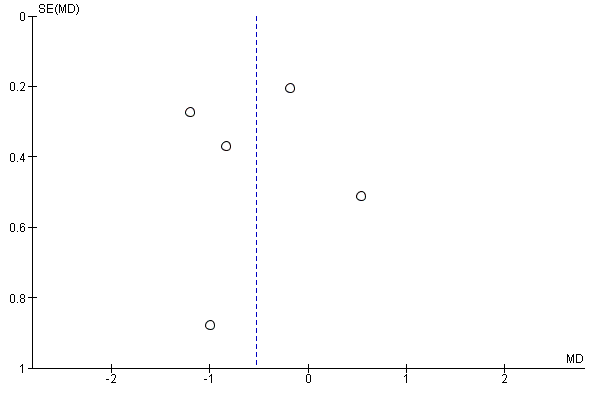 |
| --- | --- |
| **Pain scores (static) at 12-hours**  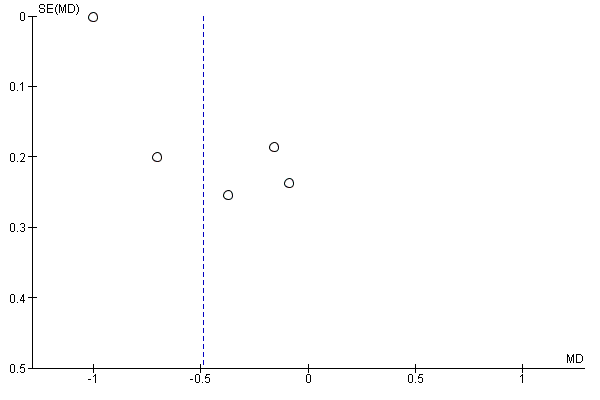 | **Pain scores (dynamic) at 12-hours**  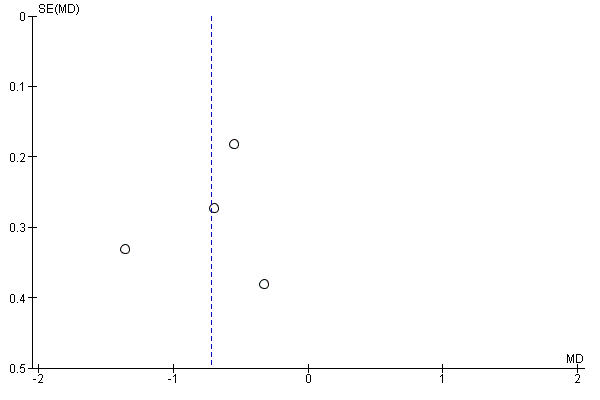 |
| **Pain scores (static) at 24-hours**  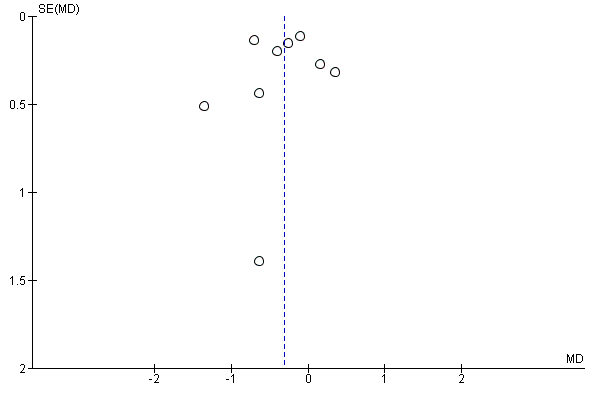 | **Pain scores (dynamic) at 24-hours**  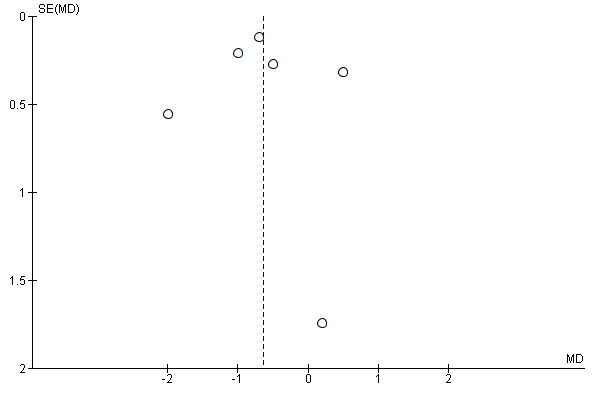 |
| **Pain scores (static- subgroup) at 24-hours**  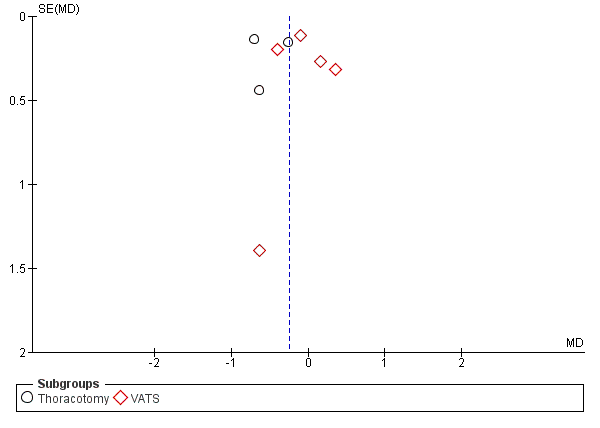 | **24-hour postoperative oral morphine (mg) equivalent consumption**  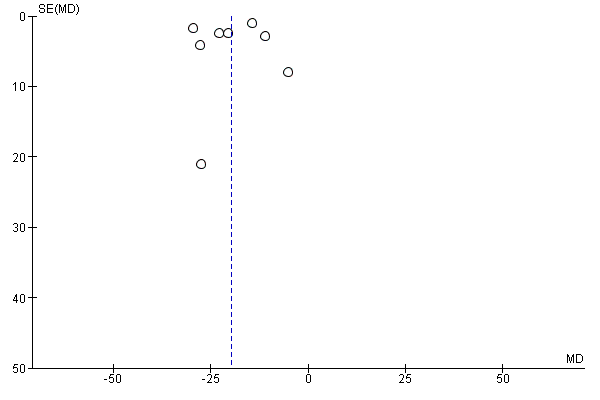 |
| **24-hour postoperative oral morphine (mg) equivalent consumption (subgroup)**  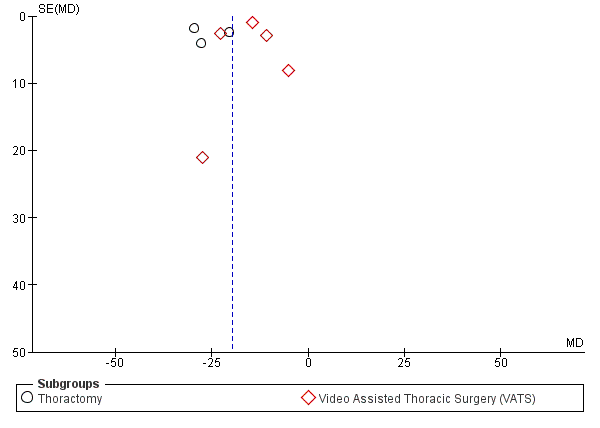 | **Time to request the first dose of postoperative analgesia.**  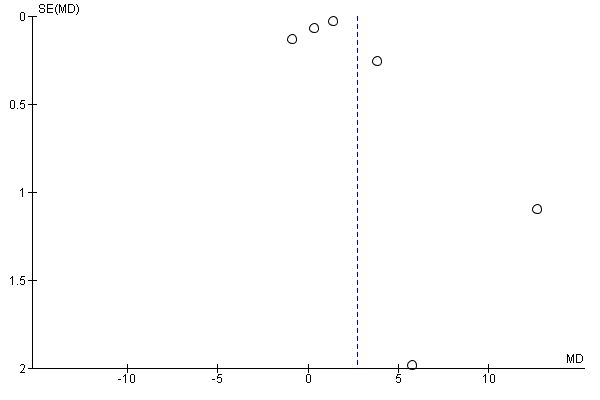 |
| **Time to request the first dose of postoperative analgesia (subgroup)**  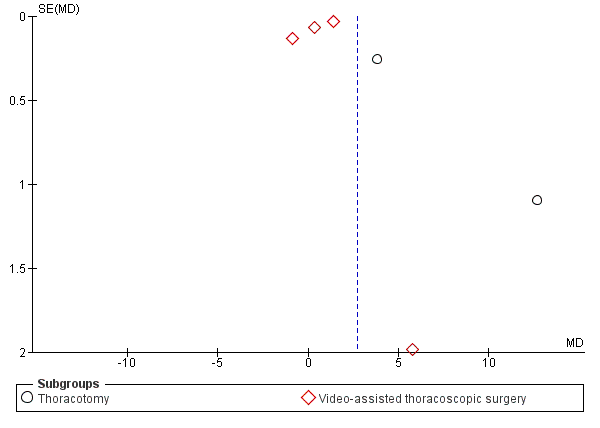 | **Successful block in first attempt**  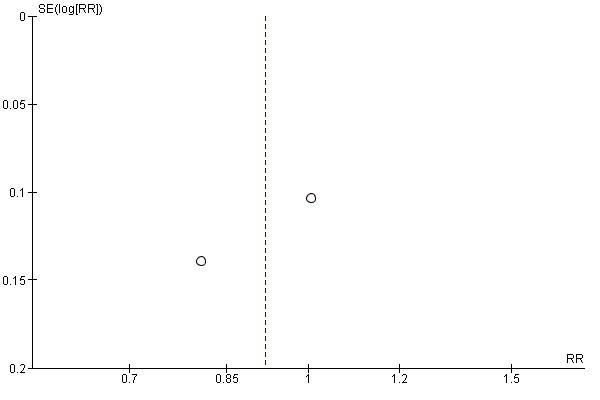 |
| **Postoperative nausea**  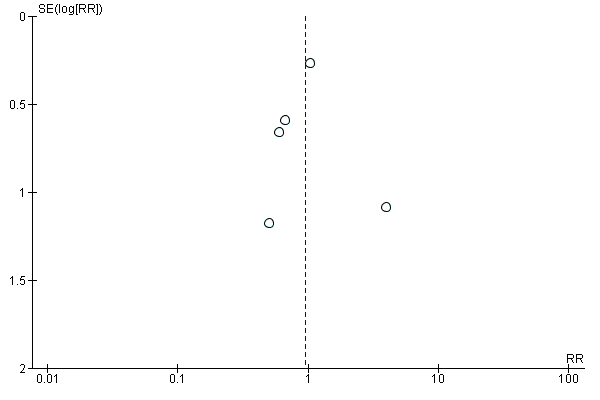 | **Postoperative vomiting**  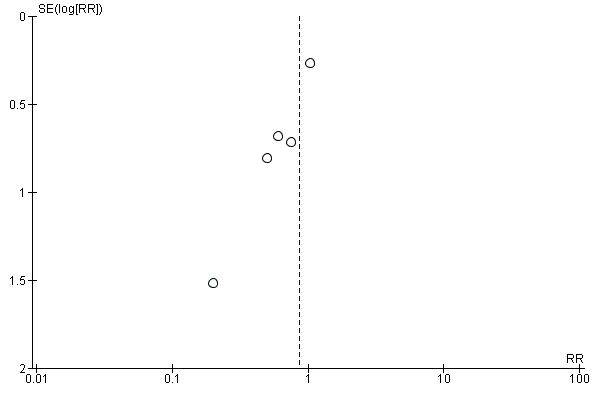 |
| **Postoperative hypotension**  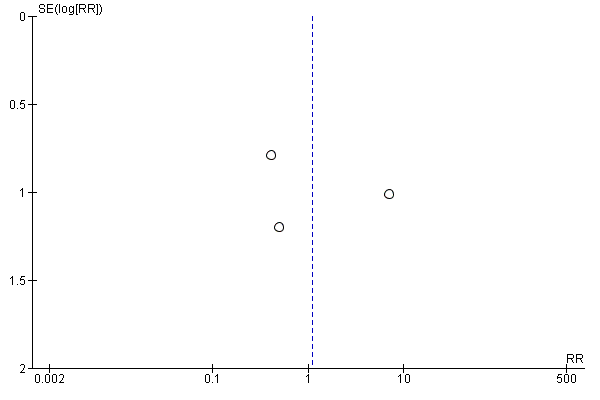 |  |

**Funnel plots of coprimary and secondary outcomes**

| **Outcomes** | ***t*-value** | ***p*-value** |
| --- | --- | --- |
| Postoperative pain scores (static) |  |  |
| - 2-hours | 6.00 | 0.81 |
| - 12-hours | 4.58 | 0.02 |
| - 24-hours | 0.29 | 0.77 |
| Postoperative pain scores (dynamic) |  |  |
| - 2-hours | 0.07 | 0.94 |
| - 12-hours | 0.52 | 0.65 |
| - 24-hours | 0.17 | 0.87 |
| 24-hour postoperative oral morphine (mg) equivalent consumption | 0.42 | 0.68 |
| Time to request the first dose of postoperative analgesia | 0.73 | 0.50 |
| Successful block in the first attempt | - | - |
| Postoperative nausea | 0.13 | 0.91 |
| Postoperative vomiting | 7.3 | 0.005 |
| Hypotension | 0.34 | 0.79 |

.

**Table- Egger’s regression**
