# Supplementary material for: Analgesic efficacy and safety of erector spinae versus serratus anterior plane block in thoracic surgery: a systematic review and meta-analysis of randomized controlled trials
Source: J Anesth Analg Crit Care. 2024 Jan 12;4:3. doi: 10.1186/s44158-023-00138-y (PMC10785351; doi:10.1186/s44158-023-00138-y)
Supplement: Supplementary file 1 — Additional file 1. Search strategy table. Table S1. Demographic characteristics of included participants. Table S2. Conversion of opioid consumption doses in 24 h to oral morphine (mg) equivalent doses. Table S3. Coprimary outcomes of the included studies. Table S4. Secondary outcomes of the included studies. Fig. S1. Funnel plots of coprimary and secondary outcomes. Table S1. Egger’s regression. Table S1. Meta-regression of coprimary outcomes [file 44158_2023_138_MOESM1_ESM.zip › Online supplementary appendix D.docx]

**ONLINE SUPPLEMENTARY APPENDIX D**

Postoperative Pain scores (static) at 24 hours

| **Covariates** | **Coefficient(β)** | **Std. Error** | **95% CI** | **p-value** |
| --- | --- | --- | --- | --- |
| Mode of Analgesia | 0.07 | 0.48 | - 1.46, 1.60 | 0.89 |
| Dose of LA | -0.06 | 0.02 | - 0.09, - 0.02 | 0.00 |

Postoperative oral morphine (mg) equivalent consumption in 24 hours

| **Covariates** | **Coefficient(β)** | **Std. Error** | **95% CI** | **p-value** |
| --- | --- | --- | --- | --- |
| Mode of Analgesia | - 7.17 | 7.86 | - 28.98, 14.64 | 0.41 |
| Dose of LA | - 0.81 | 0.58 | - 2.22, 0.59 | 0.21 |

**Table- Meta-Regression of coprimary outcomes**

LA: Local Anesthetic, Std: Standard, CI: Confidence interval
